# Training for pediatric cannot intubate cannot oxygenate: surgical airway should replace needle cricothyrotomy

**DOI:** 10.3389/femer.2026.1772381

**Published:** 2026-03-16

**Authors:** Allison M. B. Lehman, Paul Amstutz, Jackson E. Moore, Matthew Johnson, Christopher Obersteadt, Dominique Williams, Mary J. Waxman, Morgan Blubaugh, Anaya Parikh, Timothy R. Walsh, Daniel E. Bruegger, Shawn B. Sood, Adrienne N. Malik, Andrew Pirotte

**Affiliations:** 1 University of Kansas School of Medicine, Kansas City, KS, United States; 2 Mayo Clinic, Department of Emergency Medicine, Rochester, MN, United States; 3 Department of Emergency Medicine, University of Kansas Health System and University of Kansas Medical Center, Kansas City, KS, United States; 4 Department of Emergency Medicine, Denver Health Medical Center, Denver, CO, United States; 5 Department of Anesthesiology, Pain, and Perioperative Medicine, University of Kansas Health System and University of Kansas Medical Center, Kansas City, KS, United States; 6 Department of Otolaryngology, University of Kansas Health System and University of Kansas Medical Center, Kansas City, KS, United States; 7 Division of Pediatric Critical Care, University of Kansas Health System and University of Kansas Medical Center, Kansas City, KS, United States; 8 Department of Cardiology, Saint Luke’s Hospital NIH T32 MAHI Outcomes Fellow, Kansas City, MO, United States

**Keywords:** airway, CICO, cricothyrotomy, emergency front of neck access, intubation, pediatric airway emergency, pediatric intubation, surgical airway

## Abstract

Failed pediatric endotracheal intubation, prompting surgical airway management via emergency front of neck access (eFONA), is an intrinsically challenging topic. While rare, all emergency airway providers must be equipped to handle these complex clinical scenarios, both through initial training and maintenance of competency. Needle cricothyrotomy is a commonly taught method in cannot intubate cannot oxygenate (CICO) scenarios for children under age eight. However, needle cricothyrotomy is technically difficult, profoundly rare, and maintaining competency is a challenge. From a multidisciplinary perspective, we examine the utility of continuing to teach needle cricothyrotomy and explore alternatives. This project group suggests that pediatric eFONA training across disciplines should focus on surgical tracheotomy rather than needle cricothyrotomy, with consideration given to specialty-specific human performance factors.

## Introduction

Emergency Front-of-Neck Access (eFONA) procedures are employed in cannot intubate cannot oxygenate (CICO) scenarios. These critical airway scenarios evolve when the larynx or upper airway cannot be accessed through the oral or nasal route, and adequate alveolar oxygen cannot be delivered. The complexity and rarity of pediatric CICO scenarios have led to uncertainty regarding best practices in management (1, 2). When an adult patient cannot be adequately mask-ventilated or intubated, cricothyrotomy has been utilized to establish an emergent airway (2, 3). In children under age 8, however, emergent surgical airway management is complicated by incomplete development of the cricothyroid membrane, diminutive size of anatomic landmarks, and rarity of the procedure (4, 5).

Procedural instruction for pediatric eFONA has commonly focused on needle cricothyrotomy (6). Needle cricothyrotomy is a percutaneous surgical airway technique in which an over-the-needle catheter is passed through the cricothyroid membrane, followed by oxygenation via jet ventilation (6, 7). While needle cricothyrotomy can be a life-saving temporizing measure, it is technically challenging and profoundly rare (8, 9). Human performance factors are an important consideration for intervening in such rare, high-stakes scenarios (10–12). A 2024 editorial by Dare et al. (13) presents an ethical exploration of the fundamental challenge of this situation. When presented with an infant CICO scenario, airway-trained providers face a time-sensitive ethical dilemma: Perform an eFONA procedure despite feeling inadequately prepared and risk causing additional harm to the child or forego attempting eFONA and risk contributing to the child’s demise. The authors contend acting outside of traditional scope is ethically justified in such a situation, while acknowledging that unsuccessful attempts may increase a clinician’s sense of culpability or even prevent them from performing the procedure in the first place.

There are many barriers to developing and maintaining proficiency in pediatric eFONA. There is significant risk of procedural complications as well as inherent human factors influencing performance. This discussion examines best practices in pediatric eFONA and proposes alternative strategies from the perspective of a multidisciplinary team of pediatric airway managers. The authors recommend that pediatric emergency surgical tracheotomy be a focus of comprehensive airway skills training.

## Background

### Frequency of pediatric CICO and eFONA

The CICO scenario and ensuing need for emergency surgical airway management is rare. Quantifying frequency is difficult, with estimates ranging from 1 in 500 to 1 in 50,000 airway management events (14–17). Overall, these events are more frequent in adults than children, and more common in prehospital settings and emergency departments (EDs) than operating rooms (3, 14, 18). Estimated incidence of in-hospital failed pediatric endotracheal intubations from multicenter studies is 0.08–2% (11, 19). Severe pediatric airway complications in the acute care setting are associated with younger age and smaller size, with many cases occurring in infants <10 kg (11, 14, 20). Notably, surgical or failed airway is more likely in children with an unanticipated difficult airway (11).

For many airway providers, pediatric airway management represents a small proportion of procedural volume (8, 18, 21, 22). Pediatric CICO is a very rare complication for which airway managers must nevertheless be prepared. Approximately 85% of children receiving emergency care in the United States (US) present to general EDs, though pediatric patients represent a small share of critical procedures (18, 21). Pediatric endotracheal intubations comprise 5.4% of all intubations by US emergency physicians (12). Pediatric patients represent 3.6% of emergency surgical airways performed in the setting of ED trauma resuscitation (18).

Prehospital pediatric endotracheal intubation is also infrequent, with higher failure rates than in adults (23, 24). The National Association of Emergency Medical Services (EMS) Physicians emphasizes non-invasive airway management in pediatrics (24). A five-year retrospective review of US EMS records showed that only 2% of patients requiring advanced airway management techniques were aged 10 and under (25).

Pediatric CICO events and eFONA are exceedingly rare. A retrospective review of US pediatric EMS data showed an eFONA incidence of 0.1/1,000 in the prehospital setting (26 cases which did not distinguish technique) (17). In one multicenter study of currently practicing general and pediatric emergency medicine (EM) physicians, lifetime performance rate for pediatric needle cricothyrotomy was 6.8% (8). Of 2,446 senior EM physicians at 96 sites worldwide, 1.2% reported performing or supervising any pediatric surgical airway in the past year (8). Of these surgical airways, 5 used needle and 8 used open technique (8). A single three-year study of pediatric airway management by air transport reported a 4.2% rate of rescue techniques, including one needle cricothyrotomy out of 260 pediatric intubation attempts (26). The fourth National Audit Project (NAP4) in the United Kingdom identified 5 pediatric cases necessitating emergency surgical airway; one unsuccessful cricothyrotomy, three tracheotomies, and one child who died during transfer for tracheotomy (14).

### Pediatric and adult emergency surgical airway guidelines

In 2011, NAP4 suggested a need for improved preparation and training for airway complications (14, 27). Since then, expert guidance for airway management has expanded. Guidance for CICO management varies, particularly for pediatrics (6, 12, 28–31).

In 2015, the Difficult Airway Society (DAS) published guidelines for unanticipated difficult airway in a child age 1 to 8, advising that procedural approach to the CICO patient should depend upon availability of an otolaryngologist (ENT) for assistance (29). If ENT is unavailable, needle cricothyrotomy with pressure-limited jet ventilation is recommended as first-line technique, followed by surgical cricothyrotomy or tracheotomy if needle cricothyrotomy fails (29).

The All India Difficult Airway Association (AIDAA) 2025 guidelines for the management of unanticipated difficult airway in pediatrics recommend tracheotomy as the preferred method for emergency surgical airway in children ≤12 when trained surgical assistance is available (30). AIDAA prefers a scalpel-bougie-tube technique (30). In the absence of surgical assistance, AIDAA advises cricotracheal needle puncture in children <5 and needle cricothyrotomy in children 5–12 (30).

The 2024 European Society of Anaesthesiology and Intensive Care (ESAIC) and British Journal of Anaesthesiology (BJA) joint guidelines for airway management in neonates and infants recommend surgical tracheotomy as first-line eFONA (12). These guidelines state that “surgical cricothyroidotomy and percutaneous needle cricothyroidotomy are not suitable options in neonates and infants; for the former because the small size of the cricothyroid membrane will likely render insertion of a tracheal tube impossible, and for the latter because of the unfavorable anatomy” (12).

The DAS recently updated guidelines for adult eFONA (defined as patients age >8 years) (32). In 2015, DAS guidelines recommended a scalpel rather than a cannula approach (33). DAS 2025 guidelines specify a scalpel-bougie-tube technique using a vertical skin incision (32). However, they include a caveat that airway managers should train in whichever scalpel eFONA technique is likely to be most successful in their hands (32).

The American Society of Anesthesiologists (ASA) 2022 difficult airway algorithms for both pediatrics and adults recommend: “The airway manager’s assessment and choice of techniques should be based on their previous experience, available resources (including equipment), availability and competency of help, and the context in which airway management will occur” (28). Recommended eFONA techniques include surgical cricothyroidotomy, needle cricothyroidotomy with a pressure-regulated device, large-bore cannula cricothyroidotomy, or surgical tracheotomy (28). ASA also suggests considering rigid bronchoscopy or extracorporeal membrane oxygenation (ECMO) (28).

## Procedural considerations

### Needle cricothyrotomy

Needle cricothyrotomy is traditionally used in pediatric CICO due to small airway size, quick access, and the potential to minimize tissue trauma from catheter insertion (1). Needle cricothyrotomy has also been utilized due to physician reluctance to perform surgical airways in children (1, 33, 34). When successful, it is a temporizing oxygenation measure requiring low frequency transtracheal jet ventilation (3, 7, 29, 35). Once a cannula is introduced into the trachea, its hub is attached to high flow oxygen using an adapter to allow for intermittent oxygen insuffiation (7). Needle cricothyrotomy does not provide adequate ventilation and must be replaced with a definitive airway, ideally within 40 min (3, 7, 30). The risk of barotrauma with jet insuffiation is significant, particularly with an obstructed airway, as a marginally patent airway is necessary for exhalation (7, 36, 37). Other complications include pneumothorax, air embolism, bleeding, and subcutaneous emphysema (which may further obscure anatomic landmarks) (34, 35, 38, 39). Device-related complications include kinking, breaking, and dislodgement from the airway or oxygenation device (35).

Reported failure rates of needle cricothyrotomy range from 36–63% (38, 39). Challenging anatomy and identification of the cricothyroid membrane likely contribute (23, 34, 35, 38). In a neonate, the cricothyroid membrane is only 2.6 ± 0.7 mm by 3 ± 0.63 mm (40). One study showed that pediatric anesthesiologists accurately identified the cricothyroid membrane by palpation in infants and children 1–8 years old only 29% of the time (4). Additionally, the ideal angle of needle insertion is 45 degrees or less from a caudal approach, which is difficult to achieve without hyperextension of the child’s neck (34, 37).

Interestingly, point of care ultrasound (POCUS) may improve accuracy of anatomic landmark identification. The cricothyroid membrane can be accurately identified on ultrasound in 30 s to 1 min, comparable to time to identify via palpation (4, 41). POCUS is often available in ED resuscitation settings, but its use should not delay time to airway. Ultrasound-guided cricothyroid palpation practice may also improve landmark identification (42, 43).

### Surgical tracheotomy

Surgical tracheotomy is the primary alternative to needle cricothyrotomy for pediatric eFONA (12, 28–30). Surgical tracheotomy creates a definitive airway, allows for adequate oxygenation, and provides ventilation using standard equipment (3). Given the rarity of pediatric eFONA, variety of practice, and lack of centralized global reporting, there is no established comparison of techniques in pediatrics (38). A systematic review of emergency pediatric surgical airway techniques in animal models reported an 88% scalpel tracheotomy success rate with 38% complication rate (38). This is comparable to pooled global adult prehospital tracheotomy success rates of 90.5–92% (compared to 52–65.8% success for needle cricothyrotomy) (44, 45).

Risks of surgical tracheotomy include (but are not limited to): bleeding, injury to the thyroid and surrounding structures, and tracheal injury potentially complicated by tracheal stenosis with incision of >2 tracheal rings (34). However, these risks should be weighed against the high failure and complication rates of needle cricothyrotomy, particularly if the airway is completely obstructed (38). Cricothyrotomy carries a risk of subglottic stenosis (2%) and dysphonia (50%), whereas tracheotomy largely avoids these morbidities by bypassing the subglottic region (46). A single center 30-year study found a pediatric tracheotomy-related complication rate of 23% and mortality rate under 1% (47).

There are multiple techniques for pediatric emergency surgical tracheotomy. Simpson and colleagues developed a technique for emergent pediatric surgical tracheotomy intended to be quick, avoid common procedural pitfalls, and use equipment readily available in EDs (5). The technique is as follows:

“[A] midline vertical incision is made through the skin overlying the trachea. After using palpation to confirm tracheal location, a single 0-silk stay suture is placed in the sagittal plane in the midline through the overlying soft tissue and the trachea. Both sides of the suture are then pulled upward and held firmly by an assistant while the primary operator dissects the overlying soft tissue and exposes the trachea. Then, a horizontal incision is made in the trachea superior to the stay suture… then a bougie is inserted into the trachea, followed by an endotracheal tube” (5).

Berger-Estilita and colleagues offered a different five-step protocol for pediatric emergency surgical tracheotomy, using a vertical midline incision, retraction with Backhaus towel clamps, vertical tracheal incision using sharp-pointed scissors, and insertion of an endotracheal tube (34).

### Developing and maintaining proficiency in pediatric eFONA

Technical skill proficiency is an important aspect of preparation for this high-stakes, uncommon scenario. For novice trainees, repetition is necessary to develop proficiency (48). Among EM residents, scalpel-bougie-tube cricothyrotomy speed continued to increase after three attempts (49). When cricothyrotomy simulation was completed annually for three years, mean procedure times became faster with each year of experience, and the number of attempts to maximize speed decreased to saturation by year three (49).

Maintaining proficiency is also challenging. In an international survey assessing emergency physician confidence in critical airway procedures, 8.1% of respondents felt confident performing a pediatric surgical airway, correlated only with frequent hands-on practice (8). For attending anesthesiologists, skill retention for percutaneous or bougie cricothyrotomy has been demonstrated 12 or 15 months after training sessions, though another study suggested skill decay as early as three months after brush-up training (48, 50, 51). These findings suggest that annual hands-on eFONA refresher training may be needed to maintain proficiency.

It is unknown whether adult eFONA training experience translates well to pediatric techniques (12). Strategies to assess this issue have been pursued in previous research. Animal models have been used to teach pediatric needle cricothyrotomy and surgical tracheotomy (22, 38, 52). High fidelity 3D printed airway models have also been used (53). However, the unpredictable, high stress environments of real clinical scenarios leading to CICO are not well replicated in simulation or models (1, 14, 54).

### Human factors

Performance in emergent scenarios depends on non-technical skills such as situational awareness, communication, teamwork, and decision making. However, these skills are susceptible to deterioration under stress (1, 32, 54). Cognitive overload can result in fixation, omission, and failure to act (55). In difficult airway scenarios, cognitive anchoring on upper airway rescue may have catastrophic outcomes (1, 54, 56).

Human factors is a discipline focused on facilitating “doing the right thing” by understanding and designing processes and systems which optimize human wellbeing and performance under real conditions (10, 57). Foundational tools (e.g., guidelines, protocols) help professionals prepare for an event, and implementation tools (e.g., algorithms, checklists, equipment kits) reduce cognitive load and improve outcomes (55, 58, 59).

Pediatric airway management arguably has an inherent increased cognitive load, given the patient population requiring intervention. Regardless of clinician experience or competency, performance may be compromised under the stress of ongoing failed airway attempts. Algorithms that require a provider to choose between techniques or request assistance (which may not be available in every setting) may result in cognitive overload. Some cognitive tools for airway management specifically address this barrier to success (12, 28, 54, 58). By encouraging active transition to eFONA rather than recommending an abrupt change in overall approach, these tools help bypass anchoring and fixation (54, 55).

## Recommendations for practice

Although airway emergencies are less frequently encountered in children than adults, airway providers must be competent to manage pediatric CICO scenarios (14, 16, 21, 22). In many cases, the proceduralist performing a pediatric surgical airway may be doing so for the first and only time in their career (21, 54, 60).

In these harrowing airway scenarios, the most important factors to consider are efficacy and speed (60). Upper airway obstructions are a known cause of hypoxemia, which if not treated quickly can lead to cardiac arrest and anoxic brain injury. The younger and smaller the patient, the faster hypoxemia can develop (20, 61). Aggressive preoxygenation should be attempted, but may have limited effect if a child’s airway is obstructed upon airway provider arrival.

Needle cricothyrotomy is one option for pediatric surgical airway; however, success is dependent on a variety of factors. These include anatomic variations, jet ventilation equipment, procedural skill, and human performance factors (3–5, 23, 34, 35). Needle cricothyrotomy cannot be performed optimally in many settings (3, 23, 36). Even successful needle cricothyrotomy still carries significant risk of barotrauma and severe complications which may lead to lifelong disability. Surgical tracheotomy provides a definitive airway with higher first-time success rates (38, 44, 47). Additionally, surgical tracheotomy offers a lower risk of airway injury and is anatomically appropriate for the pediatric population (considering challenges associated with a small cricothyroid membrane) (38).

If needle cricothyrotomy fails or is not procedurally pursued, surgical airway should be completed. Additionally, needle cricothyrotomy is a temporizing measure only, and must be converted to tracheostomy to allow ventilation (35). We find limited use for needle cricothyrotomy, but in settings where POCUS, jet ventilation equipment, and a timely surgical consult are readily available, it may remain a consideration.

Multidisciplinary guidance has moved toward scalpel cricothyrotomy in patients age >8. The Difficult Airway Society recommends a single pathway for eFONA in age 8 and above: scalpel-bougie-tube cricothyrotomy with a vertical skin incision (32). In addition to efficacy, this technique is recommended due to difficulty of cricothyroid palpation, reduced choice burden, and relative simplicity to perform with widely available equipment (32). In the US prehospital setting, the National Association of EMS Physicians recommends a scalpel technique due to higher efficacy (31).

Given physicians’ lack of confidence in managing pediatric surgical airway, there is need for improvement in proficiency maintenance (21, 56). Training for a once-in-a-lifetime procedure must be efficient and high yield.

From a multidisciplinary perspective, we suggest that a scalpel technique should be taught by airway management training programs regardless of patient age. Multiple pediatric difficult airway guidelines prefer surgical tracheotomy in a CICO scenario if a trained provider is available (12, 29, 30). AIDAA recommends a scalpel-bougie-tube tracheotomy technique (30). Simpson’s scalpel-bougie-tube emergent pediatric surgical tracheotomy approach is similar to the DAS adult cricothyrotomy technique (5, 33). Teaching and reviewing the two techniques together could be an efficient use of training time to maintain proficiency for a rare procedure.

These authors recognize that every pediatric CICO scenario is nuanced and patient specific. Additionally, each institution and airway practitioner have different resources and areas of expertise. Patient size, physiology, anatomy, disease processes, rapid availability of surgical colleagues, ECMO capability, concurrent interventions, utilization of apneic oxygenation, availability of paralytic reversal, and other temporizing measures may all influence airway management choices. Significant psychological barriers to recognizing and promptly intervening in a CICO situation also impact clinical decision making (2, 54, 56, 62). As such, our endorsement of surgical tracheotomy is a recommendation, not a mandate. Airway managers should use clinical gestalt and experience to choose the most appropriate management in each individual case. Our goal is to lower barriers to timely and effective intervention. It is our belief that efforts to standardize the procedural approach to pediatric CICO scenarios would allow for more effective training and communication so practitioners can confidently act when faced with these rare and challenging circumstances.

## Conclusion

Pediatric surgical airway management is a complex procedural topic. Many perspectives and troubleshooting methods have been suggested. Given the complex cognitive and procedural considerations inherent to pediatric surgical airway scenarios, simplicity and reproducibility are key. Surgical tracheotomy offers a definitive airway with a favorable success rate and lower risk of complications. Additionally, emergency surgical tracheotomy is procedurally similar to the surgical cricothyrotomy preferred in a CICO scenario for patients >8. With these considerations in mind, in pediatric CICO scenarios, these authors suggest that eFONA training across disciplines should transition away from needle cricothyrotomy in favor of surgical tracheotomy for patients under 8 years of age.

## Data Availability

The original contributions presented in the study are included in the article/supplementary material, further inquiries can be directed to the corresponding authors.
